# Improvement of glucocorticoid sensitivity and attenuation of pulmonary allergic reactions by exogenous supplementation with betaine in HDM and LPS‐induced allergic mouse model

**DOI:** 10.1002/clt2.70039

**Published:** 2025-02-08

**Authors:** Qing Wang, Wen He, Yufeng Zhou, Yun Liu, Xiaoling Li, Yingwen Wang, Jiayu Wang, Xiao Han, Xiaobo Zhang

**Affiliations:** ^1^ Department of Respiratory Medicine Children's Hospital of Fudan University Shanghai China; ^2^ International Co‐laboratory of Medical Epigenetics and Metabolism Ministry of Science and Technology Institute of Pediatrics Children's Hospital of Fudan University, and the Shanghai Key Laboratory of Medical Epigenetics Institutes of Biomedical Sciences Fudan University Shanghai China; ^3^ National Health Commission (NHC) Key Laboratory of Neonatal Diseases Fudan University Children's Hospital of Fudan University Shanghai China; ^4^ MOE Key Laboratory of Metabolism and Molecular Medicine Department of Biochemistry and Molecular Biology School of Basic Medical Sciences and Shanghai Xuhui Central Hospital Fudan University Shanghai China; ^5^ Department of Nursing Children's Hospital of Fudan University Shanghai China; ^6^ Guangzhou Women and Children's Medical Centre Institute of Pediatrics Guangzhou Medical University Guangzhou China; ^7^ Center for Pediatric Clinical Quality Control of Shanghai Shanghai China

**Keywords:** betaine, choline metabolism, glucocorticoid resistance, severe asthma, therapeutic targets

## Abstract

**Background:**

Childhood asthma is a heterogeneous disease that exhibits different characteristics and varying severity; however, the metabolite alterations underlying the difference in asthma severity, especially in severe asthma, are not well understood. The aim of this study was to identify the plasma metabolic profile of children with different asthma severity and explore the potential intervention targets in severe asthma and glucocorticoid resistance.

**Methods:**

Untargeted liquid chromatography mass spectrometry was utilized to analyze plasma metabolites in 54 children with mild‐to‐moderate asthma, 50 children with severe asthma and 39 healthy controls. Multivariate statistical analyses were used to explore plasma metabolic alterations that were strongly associated with asthma severity. Meanwhile, the severe allergic airway inflammation mice with glucocorticoid resistance were constructed to validate the potential therapeutic capacity of metabolites.

**Results:**

The plasma metabolic profiles of children with mild to moderate asthma and severe asthma exhibited significant alterations. The distinct plasma metabolite shifts were accompanied by functional alterations in lipid metabolism, particularly choline metabolism, glycerophospholipids and sphingolipid metabolism. 11‐cis‐retinol, LysoPC (20:4 [8Z,11Z,14Z,17Z]), and glycerophosphatidylcholine were associated with exacerbated airway inflammation and lung function. Furthermore, 2‐Hydroxyestradiol, LysoPC (18:3 [6Z,9Z,12Z]), zeaxanthin, and betaine were shifted exclusively in the severe asthma group and may serve as potential biomarkers. Subsequent in vivo studies demonstrated that betaine supplementation partially improved glucocorticoid resistance.

**Conclusions:**

Overall, children with different asthma severity displayed distinct plasma metabolic patterns. These may contribute to the difference in response to glucocorticoids in childhood asthma and could be potential targets and interventions.

## 1 INTRODUCTION

Asthma, a significant health problem in childhood, is characterized by airway hyperresponsiveness, inflammation and remodeling.^1^ This heterogeneous disease, influenced by multiple factors, exhibits considerable variability in clinical features and treatment outcomes.^2^, ^3^ Most asthma is mild‐to‐moderate and can be effectively managed with low‐ or medium‐dose inhaled glucocorticoids (ICS) combined with long‐acting beta‐agonists (LABA). However, severe asthma remains uncontrolled despite optimized treatment with high‐dose ICS‐LABA, or that requires high‐dose ICS‐LABA to prevent it from becoming uncontrolled. Recurrent exacerbations significantly compromise the patient's quality of life and increase healthcare costs.^4^ Meanwhile, the children with severe asthma represent considerable challenges in treatment due to the greatly increased healthcare costs, making an urgent need for effective treatment.^5^, ^6^ Although many studies have explored possible factors associated with differences in asthma severity or failure of glucocorticoids therapy, the underlying mechanisms and therapeutic targets remain poorly understood.^7^, ^8^

Metabolomics, the comprehensive analysis of metabolites, has provided novel insights into the pathophysiological mechanism of several diseases, especially multifactorial ones, such as diabetes,^9^ cardiovascular disease,^10^ and allergic diseases.^11^, ^12^ In addition, as a true reflection of the body's response to environmental factors, metabolomics is crucial for novel biomarker discovery and treatment modalities development.^13^ The changes in metabolites associated with asthma severity may highlight important clues to study potential mechanisms and therapeutic targets in vitro and in vivo. Metabolomics analysis in asthma has revealed alterations in lipids,^14^, ^15^ steroids, amino acids,^16^ and bile acids^17^ which are related to immune responses and oxidative stress. However, the role of plasma metabolic alterations in children with different asthma severity and glucocorticoid resistance is yet to be fully elucidated.

Here, we performed untargeted metabolomics analyses by liquid chromatography mass spectrometry (LC‐MS) to characterize the plasma metabolic profiles in children with asthma. Our study presented the distinct metabolic patterns in children with different asthma severity and identified specific metabolites significantly associated with the disease severity. Additionally, we identified metabolites related to severe asthma and validated their therapeutic potential in asthma with glucocorticoid resistance. We sought to understand the underlying pathophysiological mechanisms and discover reliable biomarkers and effective interventions for severe asthma.

## 2 METHODS AND MATERIALS

### 2.1 Study population

In this study, children with asthma were recruited from the Children's Hospital of Fudan University and divided into severe asthma group (*n* = 50) and mild‐to‐moderate asthma group (*n* = 54) from January 2021 to December 2021. Healthy children (*n* = 39) were included and matched according to age and gender. As previously reported,^18^ a dynamic cohort of children with asthma was established based on the Childhood Asthma Database. A diagnosis of asthma was based on typical symptoms and a history of ≥12% reversibility in the forced expiratory volume in one second (FEV_1_) after bronchodilator administration. Each child received standardized management provided by healthcare providers and guardians for 12 months. Follow‐up questionnaires were used to assess medication adherence, drug inhalation technique proficiency, and the response to asthma treatment. Pulmonary function tests and childhood asthma control test (c‐ACT) were conducted by specialists every 3 months to evaluate the level of asthma control. The asthma severity was assessed based on the level of treatment required to control the asthma symptoms and exacerbations recommended by the GINA guideline.^4^ In the diagnosis of severe asthma in children, specific criteria are applied following a 12‐month period of optimal treatment and effective management of asthma triggers. These criteria include: (1) C‐ACT or ACT score of less than 20 points; (2) Requiring systemic corticosteroid treatment at least 2 times or hospitalization for asthma at least 1 time within the last 12 months. All asthmatic children underwent comprehensive phenotyping, including spirometry and bronchodilator reversibility, fractional exhaled nitric oxide measurement, and total serum Ig E and blood sampling. Atopy status was defined by a positive result (≥0.34 kU L^−1^) in at least one specific immunoglobulin E (sIgE) by enzyme‐linked immunosorbent assay (ELISA). Inhalant allergens included house dust mites, cockroaches, felines, canines, and fungi. Ingestion allergens included eggs, milk, nuts, fish, shellfish, and pineapple. Serum total Ig E test results were expressed as <100 kU L^−1^, 100–200 kU L^−1^, >200 kU L^−1^, with a result of not less than 100 kU L^−1^ being considered positive. All participants provided informed consent. The Institutional Ethics Committee of Children's Hospital of Fudan University approved the study protocols.

### 2.2 Plasma metabolomics samples

For protein deposition, a mixture of ice‐cold methanol/acetonitrile was added to plasma. After vortexing and ultrasonic extraction, the mixture was allowed to stand for 10 min and centrifuged at 4°C and 13,000 rpm for 10 min. The supernatant was dried, and 300 μL of 25% methanol was added to the sample. After centrifugation at 4°C and 13,000 rpm for 10 min, 300 μL of the supernatant from each sample was filtered through 0.22 μm microfilters and transferred to the LC‐MS vial. Equal volumes of each sample were mixed to create quality control samples (QCs).

### 2.3 Plasma metabolomics analysis

Sample analysis was performed on a Dionex U3000 ultra‐high‐performance liquid chromatography (UHPLC) system equipped with a Q‐Exactive plus quadrupole‐Orbitrap mass spectrometer featuring a heated electrospray ionization (ESI) source (Thermo Fisher Scientific) at Lu‐Ming Biotech. Co. Ltd. Chromatographic separation utilized an ACQUITY UPLC HSS T3 column (1.8 μm, 2.1 × 100 mm) (Waters Corporation). The binary gradient elution was composed of solvent A (water with 0.1% formic acid by volume) and solvent B (acetonitrile with 0.1% formic acid by volume). Mass spectrometry covered a range from mass‐to‐charge ratio (M/z) 100 to 1,000 with a full MS scan resolution of 70,000 and an HCD MS/MS scan resolution of 17,500. The collision energy was adjusted to 10, 20, and 40 eV. The mass spectrometer operated with a positive spray voltage of 3800 V and a negative spray voltage of 3000 V. QC samples were injected at consistent intervals via an analytical process to compile a dataset for evaluating repeatability.

### 2.4 Plasma metabolomics data preprocessing

Raw LC‐MS data were normalized using Progenesis QI V2.3 software. Compounds were identified through precise M/z, fragments, and isotopic patterns using databases of HMDB, Lipidmaps V2.3, Metlin, EMDB, and PMDB. Missing values were imputed with half the minimum observed value. The metabolomics data were imported into R for principal component analysis (PCA), orthogonal partial least‐squares‐discriminant analysis (OPLS‐DA), and partial least‐squares‐discriminant analysis (PLS‐DA). The model’s quality was evaluated using 7‐fold cross‐validation and 200 iterations of Response Permutation Testing. Variable Importance of Projection (VIP) values derived from the OPLS‐DA model. Metabolites were identified as significant if their VIP scores were above 1.0 and *p*‐values were less than 0.05 from a two‐tailed T‐test. Pathway enrichment for the differential metabolites was conducted through KEGG IDs using Hypergeometric tests. Pearson correlation analysis was employed to examine the relationship between metabolites and clinical indicators in asthma patients. Receiver operating characteristic (ROC) curves were utilized to evaluate the accuracy of the metabolites in the validation sets.

### 2.5 Mice

The allergic airway model was established according to others previously described.^19^ In brief, female C57BL/6 mice aged 6–8 weeks were sensitized by intratracheal instillation of 50 μg house dust mite (HDM) in phosphate‐buffered saline (PBS) on days 0–2, and then challenged with 5 μg HDM in PBS on days 14–17. Lipopolysaccharide (LPS) (250 ng) and dexamethasone (1 mg/kg) were administered on days 19, 20, and 22. Mice administered with the same volume of PBS served as controls. The betaine group, designated for treatment, received 2% betaine orally. The mice were sacrificed under anesthesia on day 25. Lung tissue samples were collected for histological assessments including hematoxylin and eosin (HE) staining, periodic acid‐Schiff (PAS) staining, and neutrophil immunohistochemical staining. Based on previously published methods,^20^ lung inflammation and airway mucus secretion were assessed and scored ranging from 0 to 4. Analysis of bronchoalveolar lavage fluid (BALF) was performed as described previously.^21^ Data were obtained from the FACS Canto II flow cytometer (BD, USA) and processed with FlowJo V10 software.

### 2.6 Assessment of airway hyperresponsiveness (AHR)

Briefly, mice were anaesthetized with sodium phenobarbital (100 mg/kg) and then subsequently exposed to nebulized PBS or methacholine via the respiratory tract. AHR was measured invasively with the Fine Pointe Resistance and Compliance System (Buxco Research Systems). Airway resistance was calculated by Fine Pointe software.

### 2.7 Enzyme‐linked immunosorbent assay (ELISA)

The mice blood samples were centrifuged at 1000 g for 10 min to separate the serum. The protein level of Ig E in the serum was quantified using the ELISA kit (BioLegend) according to the manufacturer's instructions.

### 2.8 Statistical analysis

Data that were normally distributed were analyzed using Student's T‐tests for comparisons between two groups, and one‐way ANOVA was used for multiple group comparisons. Categorical data were evaluated using the Chi‐square test. Non‐normally distributed data were assessed with the Wilcoxon rank sum test. Bar values in the figures represent the mean ± SEM. The level of significance was set at *p* < 0.05.

## 3 RESULTS

### 3.1 Characteristics of the study participants

There were no statistically significant differences in age (*p* = 0.546) and gender (*p* = 0.310) between the mild‐to‐moderate asthma and severe asthma. Compared with mild‐to‐moderate asthma, severe cases were associated with a greater body mass index (BMI) (*p* = 0.045). More than 90% of children with asthma were allergic, mainly to house dust mites. Although peripheral blood eosinophils were increased in children with severe asthma compared with those with mild to moderate asthma, the fractional exhaled nitric oxide (FeNO), an indicator of airway inflammation, did not differ significantly between the two groups (*p* = 0.189). Meanwhile, the data indicated a tendency towards increased peripheral blood neutrophils and a marked elevation in the neutrophil‐to‐lymphocyte ratio (NLR) in severe asthma, suggesting a potential critical role for neutrophils alongside eosinophils in severe asthma pathogenesis. As anticipated, severe asthma showed poor symptom control compared with children with mild‐to‐moderate asthma. Indices of lung function, such as FEV1% predicted and FEV1/FVC radio, exhibited progressive decreases as disease severity increased. Moreover, indicators of small airway function, specifically the predicted MEF50% and MMEF75/25%, were notably reduced in severe asthma cases compared to mild‐to‐moderate patients (*p* < 0.01) (Table 1).

**TABLE 1:** Baseline and clinical characteristics of participants.

Item	Healthy children (*n* = 39)	Mild‐to‐moderate asthma (*n* = 54)	Severe asthma (*n* = 50)	*p* Value
Male/*n* (%)	27 (69.23%)	38 (70.37%)	34 (68%)	0.310
Age/year	7.5 ± 2.75	7.61 ± 2.45	7.12 ± 2.49	0.546
BMI, median (25th, 75th percentile)	16.77 (16.22, 17.14)	16.44 (15.03, 18.51)	17.30 (15.78, 20.89)	0.081
Atopy	–	51 (94.44%)	49 (98%)	0.666
House dust mite	–	39 (72.22%)	46 (92%)	0.019
EOS, median (25th, 75th percentile)	–	230 (145, 410)	310 (180, 502)	0.033
EOS ≥ 150 μL^−1^/*n* (%)	–	42 (77.78%)	42 (84.00%)	0.421
NEU, median (25th, 75th percentile)	–	294 (239.67, 460.70)	351 (268.5, 501.5)	0.112
NLR, median (25th, 75th percentile)	–	1.07 (0.80, 1.72)	1.40 (1.11, 2.28)	0.017
IgE > 100 (kU L^−1^)/*n* (%)	–	40 (74.07%)	45 (96%)	0.036
C‐AST or ACT	–	24 (22,24)	16 (15,17)	0.000
Pulmonary function	–	–	–	–
FVC% predicted	–	99.28 ± 12.45	95.37 ± 13.70	0.170
FEV1% predicted	–	104.31 ± 14.79	96.72 ± 15.29	0.011
FEV1/FVC (%), median (25th, 75th percentile)	–	92.88 (88.67, 104.53)	87.06 (81.53, 89.83)	0.000
FEF	–	92.87 ± 18.31	95.20 ± 16.91	0.535
FEF25% predicted	–	95.87 ± 19.70	91.45 ± 20.67	0.269
FEF50% predicted	–	92.19 ± 21.78	80.88 ± 21.26	0.008
FEF75% predicted	–	88.07 ± 28.52	71.06 ± 25.31	0.001
MMEF75/25%	–	94 (79.75, 105)	80.50 (63.25, 96.5)	0.002
FeNO	–	13 (10, 21)	17.5 (9, 33)	0.189

*Note*: Values are presented median (IQR) for BMI, EOS, FEV1/FVC (%), MMEF75/25 (%) and FeNO.

Abbreviations: BMI, body mass index; EOS, eosinophil; FEF, forced expiratory flow; FEV1, forced expiratory volume in 1 s; FVC, forced vital capacity; FeNO, fractional exhaled nitric oxide; MMEF75/25%, maximal mid expiratory flow 25%–75%; NEU, neutrophil.

### 3.2 Plasma metabolomic features in children with different asthma severity

**FIGURE 1:**
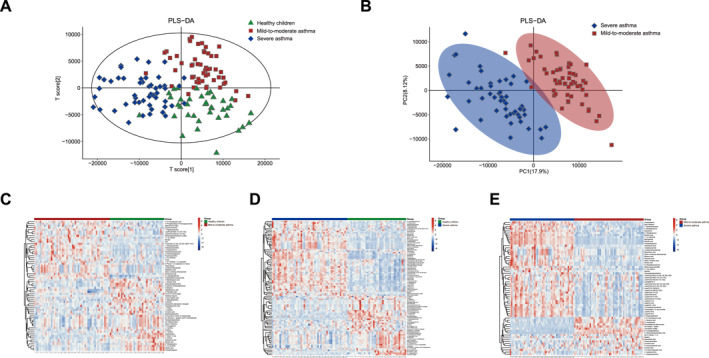
Plasma metabolomic features in children with different asthma severity. (A) PLS‐DA score plot of children with different asthma severity and healthy controls. (B) PLS‐DA score plot between children with severe asthma and mild‐to‐moderate asthma. (C–E) Representative heatmap of significantly altered metabolites in children with different asthma severity and healthy controls. VIP > 1 and *p*‐value < 0.05. PLS‐DA, partial least‐squares‐discriminant analysis.

To characterize the plasma metabolome of children with different asthma severity, untargeted metabolome profiles were generated by LC‐MS. The analysis elucidated that children with different asthma severity exhibit different plasma metabolic signatures (Figure 1A,B). Compared to healthy controls, the most discriminatory metabolites were primarily from the lipid, amino acid, and amine families, including glycerophosphocholine, lysophosphatidylcholine (LysoPC) (18:3 [6Z,9Z,12Z]), LysoPC (20:4 [5Z,8Z,11Z,14Z]), LysoPC (20:4 [8Z,11Z,14Z,17Z]), S‐lactoylglutathione, L‐proline and bilirubin (Figure 1C,D). Several metabolites, such as sphingomyelin, bile acids and phosphatidylcholine, have been found to be associated with asthma.^11^, ^12^, ^22^, ^23^ This observation highlights the pivotal role of metabolic disturbances in differences in asthma severity.

Moreover, further investigation was conducted into the plasma metabolic alterations observed in severe asthma compared with mild‐to‐moderate asthma (Figure 1E). Among the 63 identified metabolites, 16 were found to be down‐regulated in severe asthma, predominantly amino acids including 5‐aminopentanoic acid, L‐glutamic acid, and S‐lactoylglutathione. The glycerophospholipid metabolites exhibited the most significant elevation in patients with severe asthma, including LysoPC (16:0), LysoPC (17:0), LysoPC (18:0), LysoPC (18:3 [6Z,9Z,12Z]), LysoPC(20:4 [5Z,8Z,11Z,14Z]), LysoPC (20:4 [8Z,11Z,14Z,17Z]) and glycerophosphocholine (Figure 1E).

### 3.3 Abnormal alterations in sphingolipid metabolism and choline metabolism with asthma severity

**FIGURE 2:**
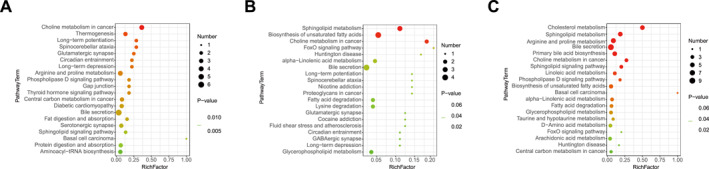
Analysis of metabolic pathways in children with different asthma severity. (A) Bubble map of differential metabolite enrichment pathways between mild‐to‐moderate asthma and healthy controls. (B) Bubble map of differential metabolite enrichment pathways between severe asthma and healthy controls. (C) Bubble map of differential metabolite enrichment pathways between mild‐to‐moderate asthma and severe asthma. The enrichment factor is the ratio of metabolites with significant differences in the pathway to the total number of metabolites. A larger enrichment factor indicates a higher enrichment. The color gradient from green to red represents a sequential decrease in *p*‐value.

Next, KEGG pathway enrichment analysis was conducted to explore the specific metabolic disruptions in children with asthma (Figure 2A–C). Compared with healthy children, 10 biological pathways were significantly perturbed in both mild‐to‐moderate asthma and severe asthma, such as choline metabolism, arginine and proline metabolism, glycerophospholipid metabolism, sphingolipid metabolism, and bile secretion. Furthermore, we observed that sphingolipid metabolism, unsaturated fatty acid biosynthesis, and choline metabolism were the most significantly altered in severe asthma compared with mild‐to‐moderate asthma (Figure 2C). Importantly, choline metabolism demonstrated notable difference between the three groups. Betaine, the main metabolite of choline, decreased significantly in severe asthma compared with mild‐to‐moderate asthma. However, the betaine was not significantly altered in children with mild‐to‐moderate asthma compared with healthy controls. This result showed that betaine may play an important role in glucocorticoid‐resistant severe asthma with glucocorticoid resistance.

### 3.4 Alterations in plasma metabolites associated with asthma severity in children

**FIGURE 3:**
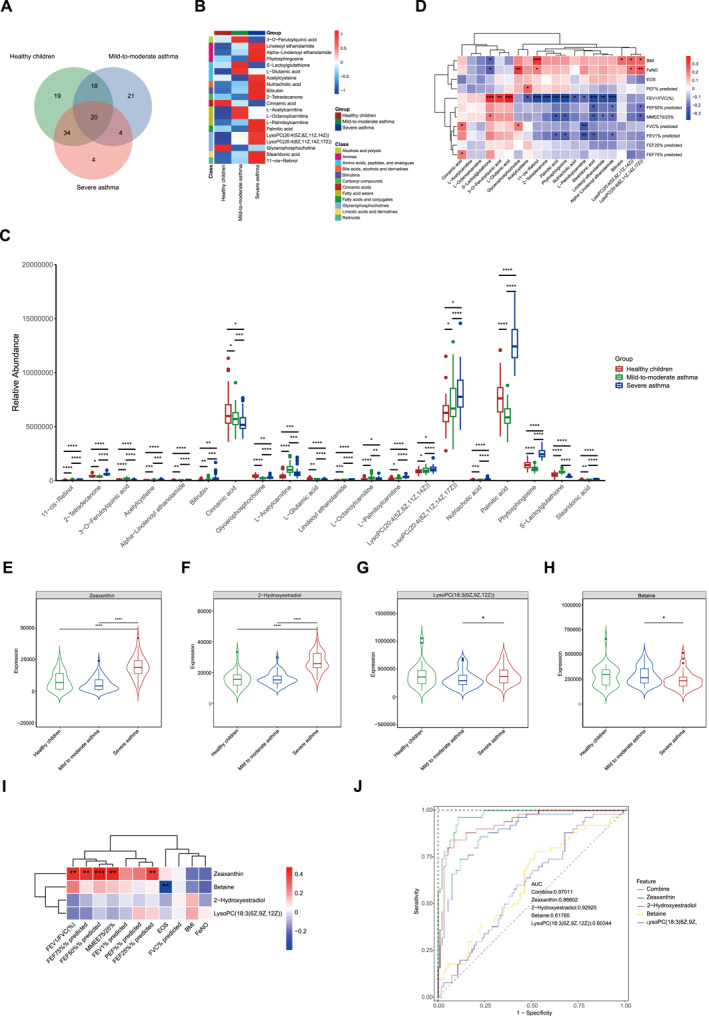
Identification of plasma metabolites associated with asthma severity in children. (A) Venn diagram displays the number of differentially expressed metabolites between the three groups. (B) Hierarchical clustering analysis of the metabolites shared by the three groups. (C) Box plot of the relative abundance of metabolites shared by the three groups. (D) Heat map of the correlation intensity between trends in metabolites with trends in clinical indicators in children with different asthma severity. (E–H) Violin plots of the relative expression of four differential metabolites that were specific in severe asthma. (I) Heat map of the correlation intensity between the four plasma metabolites and clinical indicators in severe asthma children. (J) ROC curves analysis of the four metabolites for discriminating severe asthma patients from mild‐to‐moderate asthma children and healthy children. ROC, receiver operating characteristic.

To identify metabolites associated with asthma severity, we analyzed metabolites that were differentially expressed between the three groups. Twenty plasma metabolites were significantly altered during the progression of asthma (Figure 3A). LysoPC (20:4 [5Z,8Z,11Z,14Z]), LysoPC (20:4 [8Z,11Z,14Z,17Z]), L‐palmitoylcarnitine, nutriacholic acid, linoleoyl ethanolamide, alpha‐linolenoyl ethanolamide, stearidonic acid, 11‐cis‐retinol, and bilirubin were up‐regulated with asthma severity. There were five metabolic molecules that were found to be elevated in mild‐to‐moderate asthma compared to healthy controls but decreased in severe asthma compared to mild‐to‐moderate asthma: L‐glutamic acid, 3‐O‐feruloylquinic acid, S‐lactoylglutathione, L‐acetylcarnitine, and L‐octanoylcarnitine (Figure 3B,C).

In addition, we investigated whether the above metabolites were correlated with airway inflammation and lung function impairment in asthma patients. Several metabolites were significantly associated with airway inflammation. LysoPC (20:4 [5Z,8Z,11Z,14Z]), LysoPC (20:4 [8Z,11Z,14Z,17Z]), 11‐cis‐retinol and glycerophosphocholine were positively correlated with FeNO, while S‐lactoylglutathione was negatively correlated with FeNO. Notably, plasma metabolites associated with FeNO were also correlated with BMI in children, such as LysoPC (20:4 [5Z,8Z,11Z,14Z]), 11‐cis‐Retinol and LysoPC (20:4 [8Z,11Z,14Z,17Z]). Moreover, several metabolites showed a correlation with lung function. 3‐O‐feruloylquinic acid, S‐lactoylglutathione, and L‐glutamate showed a positive correlation with FEV1/FVC (%). S‐lactoylglutathione was also positive with MMEF75/25 (%). Conversely, elevated concentrations of several lipid metabolites, such as palmitic acid, L‐palmitoylcarnitine, stearicosadioic acid, and 11‐cis‐retinol, were associated with more severe lung function impairment (Figure 3D). Collectively, our findings provided potential evidence for a correlation between the onset and severity of childhood asthma and lipid metabolism disorders and antioxidant imbalances.

### 3.5 Distinctive plasma metabolic alterations observed in children with severe asthma

To better understand the effect of plasma metabolites on severe asthma, we analyzed metabolites that were specifically altered in severe asthma. Four metabolites were found to be specially perturbed in children with severe asthma when compared to healthy controls and mild‐to‐moderate asthma: betaine, zeaxanthin, LysoPC (18:3 [6Z,9Z,12Z]), and 2‐hydroxyestradiol. Among these metabolites, betaine was down‐regulated in children with severe asthma, while the zeaxanthin, LysoPC (18:3 [6Z,9Z,12Z]) and 2‐hydroxyestradiol were up‐regulated compared with mild‐to‐moderate asthma (Figure 3E–H). In addition, we observed that the relative expression of zeaxanthin was positively correlated with lung function‐related indices, and betaine was negatively correlated with peripheral blood eosinophils (Figure 3I). The ROC curve analysis indicated that the combined diagnosis of all four substances resulted in an AUC value of 0.97 on the ROC curve, highlighting their significant role in the diagnosis of severe asthma. 2‐hydroxyestradiol has the largest area under the AUC curve of approximately 0.92, followed by zeaxanthin with an area under the AUC curve of about 0.89 (Figure 3J). However, the diagnostic value of these metabolites needs further validation in larger populations.

### 3.6 Betaine supplementation improved glucocorticoid sensitivity in severe asthma

**FIGURE 4:**
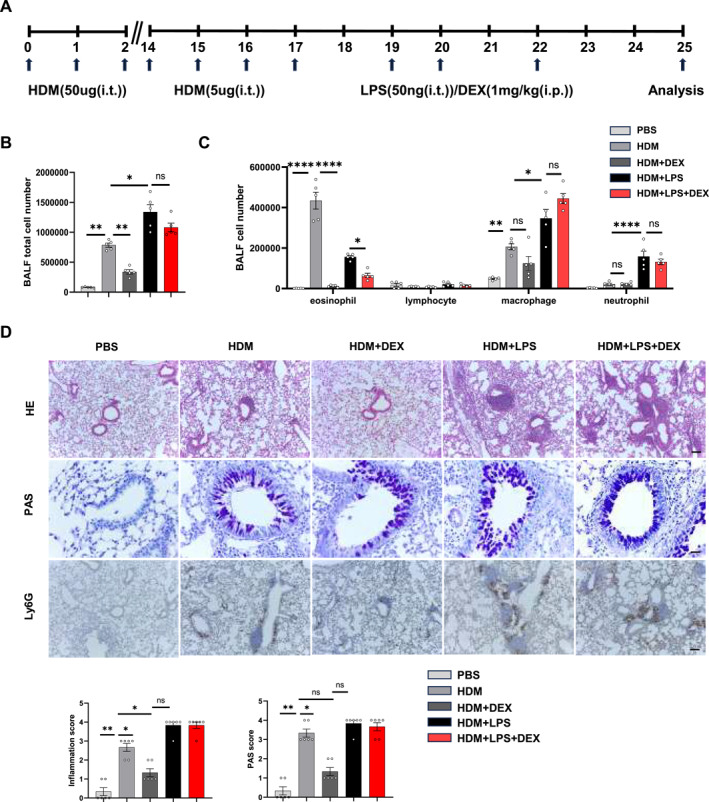
Exacerbated airway inflammation and steroid resistance in HDM and LPS induced mice. (A) Schematic diagram of the experimental design. (B) Total and differential (C) BALF cell numbers in mice with different treatments detected by flow cytometry. (D) Representative images of HE‐stained slides (Scale bar = 400 μm), and PAS‐stained slides for mucus (purple stain) in the airway (Scale bar = 100 μm). Immunohistochemistry for Ly6G to identify neutrophils in lung tissue (Scale bar = 400 μm). Inflammation score and PAS score. Data are shown as means ± SEM (*n* = 4‐6 mice/group) from 2 to 3 independent experiments. BALF, bronchoalveolar lavage fluid; HDM, house dust mite; HE, hematoxylin and eosin; LPS, lipopolysaccharide; PAS, periodic acid‐Schiff; ns, not significant. *p < 0.05, **p < 0.01, ***p < 0.001, and ****p < 0.0001.

Subsequently, we developed severe allergic airway inflammation in mice with glucocorticoid‐resistance induced by HDM and LPS based on a previous study.^19^ PBS‐sensitized and challenged mice were used as negative controls (Figure 4A).

**FIGURE 5:**
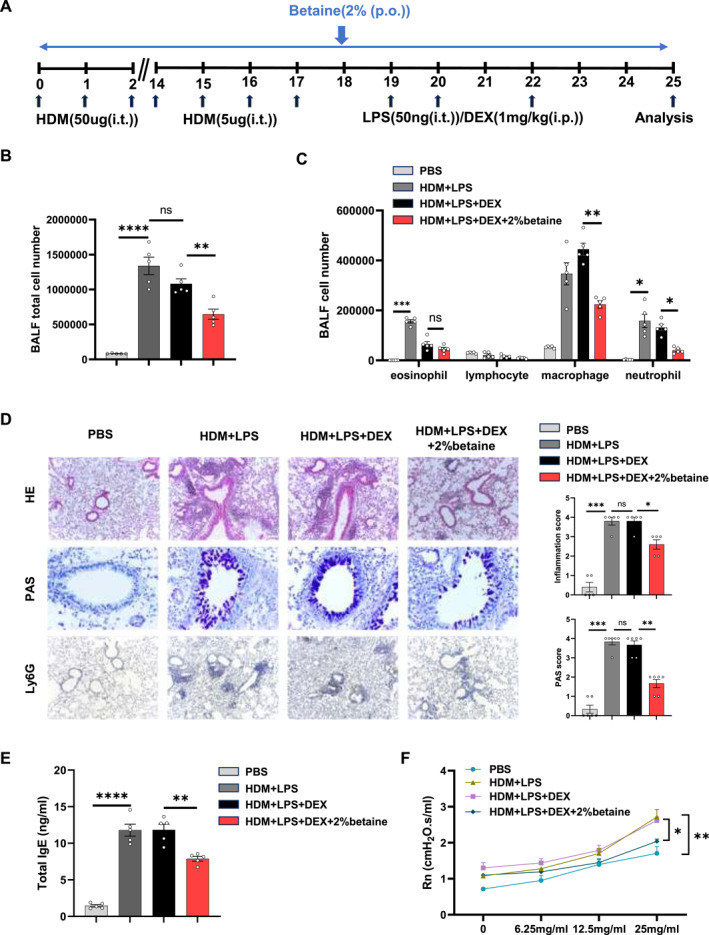
Exogenous betaine supplementation enhances glucocorticoid sensitivity in severe asthma. (A) Schematic diagram of the experimental design of exogenous betaine supplementation for the treatment of mice with severe asthma. (B) Total BALF cell numbers and differential (C) BALF cell percent or numbers in mice with different treatments detected by flow cytometry. (D) Representative images of HE‐stained slides (Scale bar = 400 μm), and PAS‐stained slides for mucus (purple stain) in the airway (Scale bar = 100 μm). Immunohistochemistry for Ly6G to identify neutrophils in lung tissue (Scale bar = 400 μm). Inflammation and PAS scores were quantified. (E) Detection of serum Ig E expression by ELISA in mice. (F) Assessment of methacholine‐induced airway hyperresponsiveness (AHR) in mice. Central airway resistance (Newtonian resistance, Rn) values are shown. Data are shown as means ± SEM (*n* = 4–6 mice/group) from 2 to 3 independent experiments. BALF, bronchoalveolar lavage fluid; PAS, periodic acid‐Schiff; ns, not significant. *p < 0.05, **p < 0.01, ***p < 0.001, and ****p < 0.0001.

Compared to the HDM group, the inflammatory response in the HDM and LPS groups was notably pronounced, with a considerable increase in total inflammatory cells with increased neutrophil and lower eosinophil counts. Histological analysis revealed a prominent lung inflammatory response characterized by neutrophil infiltration and mucus hypersecretion in HDM and LPS mice. Furthermore, treatment with dexamethasone was found to reduce the symptoms of HDM‐induced‐allergic airway disease. However, HDM and LPS mice did not show any improvement in lung inflammation or airway mucus secretion following dexamethasone treatment (Figure 4B–D). The results indicated the successful development of the severe allergic airway inflammation with glucocorticoids resistance. Next, we investigated whether administered betaine combined with dexamethasone could improve glucocorticoids treatment (Figure 5A). There was a significant reduction of the total BALF inflammatory cells in mice treated with dexamethasone combined with betaine (Figure 5B). A significant decrease was observed in neutrophils compared to dexamethasone treatment alone (Figure 5C). Moreover, betaine treated group showed a significant reduction in inflammatory cell infiltration, particularly in the proportion of neutrophils, which was consistent with the reduction in cell number of BALF. Additionally, the extensive goblet cell hyperplasia and mucus production observed in primed and challenged mice was reduced in mice treated with dexamethasone combined with betaine (Figure 5D). In addition, serum total Ig E and airway hyperresponsiveness in HDM‐ and LPS‐challenged mice were suppressed by glucocorticoids combined with betaine (Figure 5E,F). Betaine supplementation significantly attenuated the lung inflammation, which may be closely related to the suppression of non‐T2 cell inflammatory response. Notably, either with or without glucocorticoid resistance, betaine supplementation without glucocorticoids could not alleviate the symptoms of HDM‐induced‐allergic airway disease (Figure 1S in Supporting Information S1).

## 4 DISCUSSION

In this study, we described alterations in the plasma metabolome associated with asthma severity in children. The results suggest a correlation between plasma metabolites and the severity of asthma in children. Furthermore, we explored the special altered plasma metabolites in children with severe asthma. Importantly, we discovered that glucocorticoid resistance in severe asthma was associated with reduced levels of betaine in the plasma. Exogenous supplementation with betaine significantly improves glucocorticoid resistance and lung function impairment in severe asthma with glucocorticoid resistance.

The asthma patients with different severity exhibited significantly different plasma metabolite features. Compared with mild‐to‐moderate asthma, children with severe asthma showed significant disruption in sphingolipid metabolism, with upregulated levels of sphingolipid metabolites. Sphingolipids, a distinct class of lipids that include phosphosphingolipids, ceramides and sphingomyelins, are crucial for immune cell regulation and function,^24^ but their exact role in asthma is not fully understood. Our study found that Sphingolipid metabolism was most significantly upregulated in children with severe asthma compared with children with mild‐to‐moderate asthma and healthy children. The elevated sphingolipid levels could potentially drive asthma progression as higher concentrations of ceramides and sphingosine‐1‐phosphate have been observed in adults with asthma, especially in patients with uncontrolled conditions.^25^ However, previous studies on sphingolipid metabolism in early life suggest that reduced ceramides and sphingomyelins due to increased expression of the stomatocyte‐like 3 (ORMDL3) gene, which inhibits serine palmitoyltransferase (SPT), is associated with a higher risk of wheezing and early onset asthma before the age of 3 years.^22^, ^26^ We speculate that sphingolipid metabolic imbalances may play different roles at different stages of asthma development. Early in life, it is likely that genetic factors predominate in the development of asthma, followed by a combination of environmental and genetic factors.^27^ In addition, choline is important in the metabolism of phosphatidylcholine and sphingolipids.^28^, ^29^ Abnormal alterations in phosphatidylcholine metabolism and sphingolipid metabolism may contribute to abnormal choline metabolism in asthma patients.

Lysophosphatidylcholine (LPC) is a class of biologically active pro‐inflammatory lipids produced from the hydrolysis of glycerophospholipids by phospholipases. Previous research has indicated that LPC may act as a pro‐inflammatory role in asthma,^29^, ^30^ which is consistent with our findings. We discovered that LysoPC (20:4 [8Z,11Z,14Z,17Z]) and LysoPC (20:4 [5Z,8Z,11Z,14Z]) were strongly correlated with asthma severity, and higher levels of LysoPC (20:4 [8Z,11Z,14Z,17Z]) were associated with more severe lung function impairment and airway inflammation in asthma patients. Elevated LPC levels could enhance the expression of inflammatory mediators, such as interleukin (IL)‐6, IL‐1*β* and IL‐33.^31^ Furthermore, prolonged exposure of airways to LPC can cause desensitisation of *β*‐adrenergic receptors and increase the sensitivity of airway smooth muscle cells to Ca2+, resulting in airway smooth muscle contraction and reduced responsiveness to *β*‐agonists.^32^

Betaine, a product of the oxidative metabolism of choline, is an important source of variable methyl groups involved in the synthesis and metabolism of methyl donors. Several studies have demonstrated the potential therapeutic effects of betaine in diseases that are closely related to disorders of lipid metabolism, including nonalcoholic and alcoholic fatty liver disease,^33^, ^34^ atherosclerosis,^35^, ^36^ and diabetes mellitus.^37^, ^38^ Our study provided a new potential link between the plasma betaine and severe asthma with glucocorticoid resistance. Significant associations were observed between the plasma betaine and glucocorticoid resistance in severe asthma patients. Further in vivo studies preliminarily confirmed that betaine supplementation improved the treatment outcomes of glucocorticoids in severe asthma. Although asthma is usually associated with eosinophilia and T2 cytokines, some patients exhibit predominantly neutrophilic disease and lack T2 cytokines, particularly those with obesity and glucocorticoid resistance. In our study, the infiltrated neutrophils around the airway could be significantly suppressed by betaine. However, the exact mechanism remains unclear and may be closely associated with the antioxidant properties of betaine and its inhibition of inflammatory cytokine production. Activated neutrophils produce large numbers of oxygen free radicals through respiratory bursts leading to respiratory epithelial damage and respiratory hyperresponsiveness.^39^ The reduced levels of glutathione (GSH) in asthma patients, especially in severe asthma, may be closely related to the peroxidation of biofilm lipids. Betaine promotes the formation of methionine from homocysteine which exerts antioxidant effects through the synthesis of GSH.^40^, ^41^ In addition, several studies have demonstrated that betaine inhibits the transcriptional activation of nuclear factor‐κB (NF‐κB) and production of tumor necrosis factor‐ɑ (TNF‐*ɑ*) and IL‐1*β*,^42^, ^43^, ^44^ which are considered to be important chemokines for neutrophils and strongly associated with poor control of severe asthma.^45^, ^46^ However, betaine supplementation alone in vitro was not sufficient to reduce airway inflammation and mucus secretion, indicating that this anti‐inflammatory effect requires additional immunomodulatory drugs, at least for the tested concentrations.

Asthma inflammatory subtypes are categorized based on sputum eosinophil and neutrophil proportions, encompassing eosinophilic, neutrophilic, mixed granulocytic, and paucigranulocytic asthma. While induced sputum cell counts remain the benchmark for delineating inflammatory phenotypes, their accessibility is limited. As an alternative, peripheral blood cell counts in the presence of a potential non‐invasive method for quantifying inflammatory cells and identifying asthma inflammation subtypes.^47^ NLR combines neutrophils as an innate inflammatory marker with lymphocytes as an allergic inflammation regulator. In neutrophilic asthma, both blood neutrophil percentage and NLR are elevated. The current investigation revealed that pediatric patients with mild to moderate asthma predominantly exhibited eosinophilic asthma. In contrast, those with severe asthma manifested a degree of neutrophilia alongside eosinophilia. Thus, the HDM and LPS‐induced glucocorticoid‐resistant‐allergic airway inflammation model constructed in this study is consistent with this inflammatory feature to some extent.

However, our study has several limitations. Firstly, our results indicate an underlying metabolic shift in the circulation of individuals with different asthma severity. However, further analysis of the metabolic patterns and diagnostic value of the biomarkers in a larger cohort is necessary. Furthermore, additional studies are required to fully elucidate the mechanisms underlying betaine to improve sensitivity to glucocorticoids in severe asthma. It is important to acknowledge that children with mild‐to‐moderate asthma and severe asthma had been treated with different doses of glucocorticoids before plasma metabolomics was performed. Further consideration needs to be given to the impact of hormone use on metabolic processes.

In conclusion, this study provided substantial evidence to support the hypothesis that the disturbed plasma metabolites of childhood asthma are related to disease severity. This finding contributes to a more comprehensive understanding of the ways in which childhood asthma is affected by plasma metabolism and provides a basis for further research into metabolically targeted strategies for improving clinical outcomes for children with severe asthma.

## AUTHOR CONTRIBUTIONS

**Qing Wang**: Writing—original draft; Writing—review & editing; Methodology; Visualization; Data curation. **Wen He**: Writing—original draft; Data curation. **Yufeng Zhou**: Methodology. **Yun Liu**: Writing—review & editing. **Xiaoling Li**: Data curation. **Yingwen Wang**: Data curation. **Jiayu Wang**: Data curation. **Xiao Han**: Writing—review & editing; Methodology; Formal analysis. **Xiaobo Zhang**: Formal analysis; Funding acquisition; Project administration.

## CONFLICT OF INTEREST STATEMENT

The authors declare no conflicts of interest.

## Supporting information

Figure S1

## Data Availability

The data that support the findings of this study are openly available in MetaboLights at https://www.ebi.ac.uk/metabolights/, reference number MTBLS10705.
