# Supplementary material for: Improvement of glucocorticoid sensitivity and attenuation of pulmonary allergic reactions by exogenous supplementation with betaine in HDM and LPS‐induced allergic mouse model
Source: Clin Transl Allergy. 2025 Feb 8;15(2):e70039. doi: 10.1002/clt2.70039 (PMC11806522; doi:10.1002/clt2.70039)
Supplement: Supplementary file 1 — Figure S1 [file CLT2-15-e70039-s001.docx]

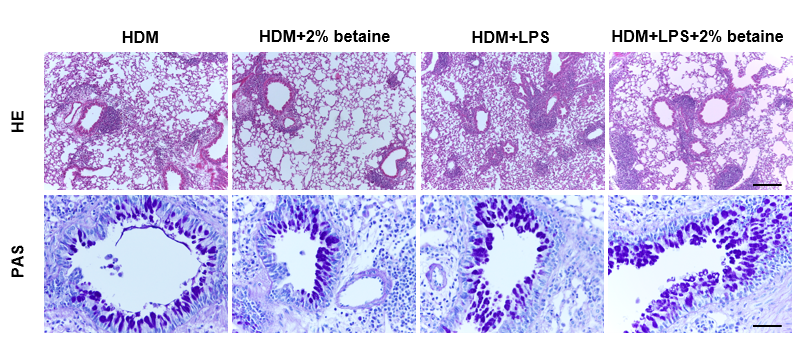


Figure 1S Exogenous supplementation of betaine alone in HDM and LPS sensitised mice. Representative images of HE-stained slides (Scale bar = 400 μm), and PAS-stained slides for mucus (purple stain) in airway (Scale bar = 100 μm).
